# A cross-country qualitative analysis of teachers’ perceptions of asthma care in sub-Saharan Africa

**DOI:** 10.1038/s41533-023-00354-7

**Published:** 2023-09-23

**Authors:** Kimesh Loganathan Naidoo, Sindisiwa Dladla, Reratilwe Ephenia Mphahlele, Gioia Mosler, Sophie Muyemayema, Andrew Sentoogo Ssemata, Elizabeth Mkutumula, Olayinka Olufunke Adeyeye, Olayinka Goodman, Yetunde Kuyinu, Rebecca Nantanda, Emmanuel Addo-Yobo, Sandra Kwarteng Owusu, Bernhard Arhin, Ismail Ticklay, Hilda Angela Mujuru, Jonathan Grigg, Refiloe Masekela

**Affiliations:** 1 Department of Paediatrics and Child Health, School of Clinical Medicine, College of Health Sciences, University of KwaZulu-Natal, Durban, South Africa; 2 Centre for Genomics and Child Health, Blizard Institute, Barts and The London School of Medicine and Dentistry, Queen Mary University of London, London, UK; 3 Child and Adolescent Health Unit (CAHU), Department of Primary Health Care Sciences, Faculty of Medicine and Health Sciences, University of Zimbabwe, Avondale, Harare, Zimbabwe; 4 Department of Psychiatry, College of Health Sciences, Makerere University, Kampala, Uganda; 5 Department of Global Health and Development, London School of Hygiene and Tropical Medicine, London, UK; 6 Malawi Liverpool Wellcome Programme, Queen Elizabeth Central Hospital, College of Medicine, Chichiri, Malawi; 7 Lagos State University College of Medicine Ikeja, Lagos, Nigeria; 8 Lagos State University Teaching Hospital, Ikeja, Lagos, Nigeria; 9 Makerere University Lung Institute, Makerere University College of Health Sciences, Kampala, Uganda; 10 Department of Paediatrics and Child Health, Makerere University College of Health Sciences, Kampala, Uganda; 11 Department of Child Health, Komfo Anokye Teaching Hospital, Kumasi, Ghana; 12 Parirenyatwa Group of Hospitals, Causeway, Harare, Zimbabwe

**Keywords:** Paediatric research, Paediatrics

## Abstract

Asthma is the most common chronic respiratory disease among school-going adolescents worldwide. However, the burden of severe asthma is highest in Sub-Saharan Africa. This study aimed to explore teachers’ perceptions of asthma care across six African countries. We conducted focus group discussions (FGDs) using a semi-structured interview guide. Interviews were audio-recorded, transcribed verbatim and analysed thematically. FGDs were conducted in Kumasi(Ghana), Blantyre (Malawi), Lagos (Nigeria), Durban (South Africa), Kampala (Uganda), and Harare (Zimbabwe) between 01 November 2020 and 30 June 2021. We identified two key themes related to asthma care; barriers to asthma care and suggestions to improve the care of adolescents with asthma. Barriers reported by teachers included a lack of knowledge and skills among themselves, adolescents, and caregivers. In addition, some traditional beliefs of teachers on asthma exacerbated challenges with asthma care in schools. Regarding suggestions, most teachers identified a need for all-inclusive asthma training programmes for teachers, adolescents and caregivers, focusing on acute episodes and mitigating triggers. Utilising teachers with personal experiences with asthma to advocate and support these initiatives was suggested. Further suggestions included the need for annual screening to enable early identification of adolescents with asthma and clarify restrictions on teachers administering asthma medications. Teachers across African schools identify multiple barriers to asthma care. Structured school education programs and annual asthma screening are key to addressing some barriers to care.

## Introduction

Asthma is the most common non-communicable disease among adolescents, affecting one in ten worldwide^1,2^. In Sub-Saharan Africa (sSA), asthma symptom prevalence in adolescents in some urban settings is as high as 21.7% and is increasing in others^2–6^. Half of the adolescents with asthma in sSA have severe symptoms, contributing to high asthma morbidity and mortality in low to middle-income countries (LMICs)^5,6^. In addition, many adolescents with asthma remain largely undiagnosed. In those diagnosed, the proportion of poorly controlled asthma remains high and is linked with poor access to appropriate asthma treatments^3–6^.

The Global Asthma Network (GAN) report has shown that 36% of adolescents with asthma suffer from severe symptoms, 36% experience symptoms with exercise and sleep disturbance due to wheezing and 41% with nocturnal cough^3,7^. Poor asthma control has been associated with poor school attendance and academic performance^8,9^. Education on asthma is viewed as a key intervention that can assist in achieving better control, especially in low-resource settings where multiple healthcare system challenges exist^7,10,11^. The school environment and the active participation of teachers in asthma care, in particular, are important for the holistic provision of care^12^.

While studies from high-income countries have noted a perceived lack of knowledge among teachers on asthma, this data is lacking in Africa^13^. However, the role of teachers in asthma care and school support systems has been noted to be poorly organised^13–15^. A recent systematic review found that having written school health policies on acute asthma and sustained asthma education programs improved preparedness to deal with acute asthma episodes amongst adolescents and improved overall asthma knowledge in teachers^16^.

Against this backdrop, there is a need for evidence of the highest quality on the barriers to achieving good asthma control and what contributes to poor asthma outcomes in urban African school-going adolescents. Adolescents spend a large part of their lives in schools where teachers hold significant leadership positions and act as role models in most African communities. This study aimed to determine the role teacher’s play in the care of adolescents with asthma and to identify barriers and facilitators to this care that are specifically related to the urban school environment in sub-Saharan African countries. We also aimed to assess the perceptions of teachers on asthma care within the school environment.

## Methods

### Study design

This study was a component of the National Institute for Health and Care Research (NIHR) funded Global Health Research Group “Achieving control of asthma in children in Africa” (ACACIA), an observational cohort study across six African countries: Ghana, Malawi, Nigeria, South Africa, Uganda, and Zimbabwe^17^. An objective of the ACACIA project was to determine the barriers to care for adolescents with asthma in the school environment. Using a Breathing Survey developed from questions in the Global Asthma Network (GAN) questionnaire, the project identified adolescents with a doctor’s diagnosis of asthma and those with symptoms of asthma but without a formal diagnosis in various schools in these countries^17^. To deepen the researchers’ team’s understanding of possible facilitators and enablers to effective asthma care and to determine possible components for a school-based asthma intervention, teachers from at least three schools in each city were selected to take part in focus group discussions (FGDs) with five to eight participants per school. The study plan, procedures, and methods were standardised across all countries^17^.

### Study population

This included teachers at the three schools selected in each city involved in the ACACIA project. The teachers selected to participate in this study were drawn from schools where both adolescents with a doctor’s diagnosis of asthma and symptoms of asthma without a diagnosis were identified. A convenience sample of teachers who consented to be included in the study were the study participants.

### Study setting

The FGDs were held between 01 November 2020 and 30 June 2021 in schools (government, private, residential, and non-residential) in cities across six countries: Kumasi (Ghana), Blantyre (Malawi), Lagos (Nigeria), Durban (South Africa), Kampala (Uganda) and Harare (Zimbabwe). The in-country team of researchers and field workers facilitated the FGDs.

### Data collection

FGDs were conducted face-to-face, with adherence to in-country-specific COVID-19 regulations. All FGDs were moderated by at least two facilitators, one of whom was a study investigator at each site. FGDs took place in English. FGDs were audio-recorded and transcribed verbatim. Discussions lasted between 40–60 minutes and followed a pre-defined question guide for all teacher FGDs (Table 1). The ACACIA core study team developed the discussion guide after multiple iterations. The underlying framework that informed the development of the discussion guide by this team was a literature review on barriers to asthma care amongst children with asthma worldwide^17^.

**Table 1:** Focus discussion group guide for teachers used in all countries.

Teacher interview guide for all FGDs across all countries	
Before the discussion	Introductions.
Discussion	1. Asthma is … (Finish the sentence).
	2. Tell us about your experience with asthma in school?
	• What would you do if a child has an asthma attack in school? • Tell us about your experiences of asthma outside of school.
	3. What do you think will improve your confidence to help a learner when they have an asthma attack (acute episode) in school?
	4. In your experience, how does asthma affect a learner in school?
	5. What are some of your ideas on how to improve life for adolescents with asthma?

### Data analysis

The transcripts from discussions were analysed using the NVivo 1.6 software program. We used an inductive analysis to examine the data^18^. A ten-member coding team was formed to standardise this analysis from across multiple countries. These ten members of the ACACIA research group from all the participating countries independently developed codes from the distributed transcripts. At least two investigators reviewed each transcript. Following this analysis, all members discussed the coding to develop a codebook using published guidelines^18,19^. This codebook was developed after multiple iterations by the ten-member coding team. A reiterative process was followed to apply the codebook to the transcribed data and determine whether further codes emerged from the team. When no new codes emerged, the codebook was assumed to be a valid representation of the data and finalised (Supplementary file 1).

**Fig. 1:**
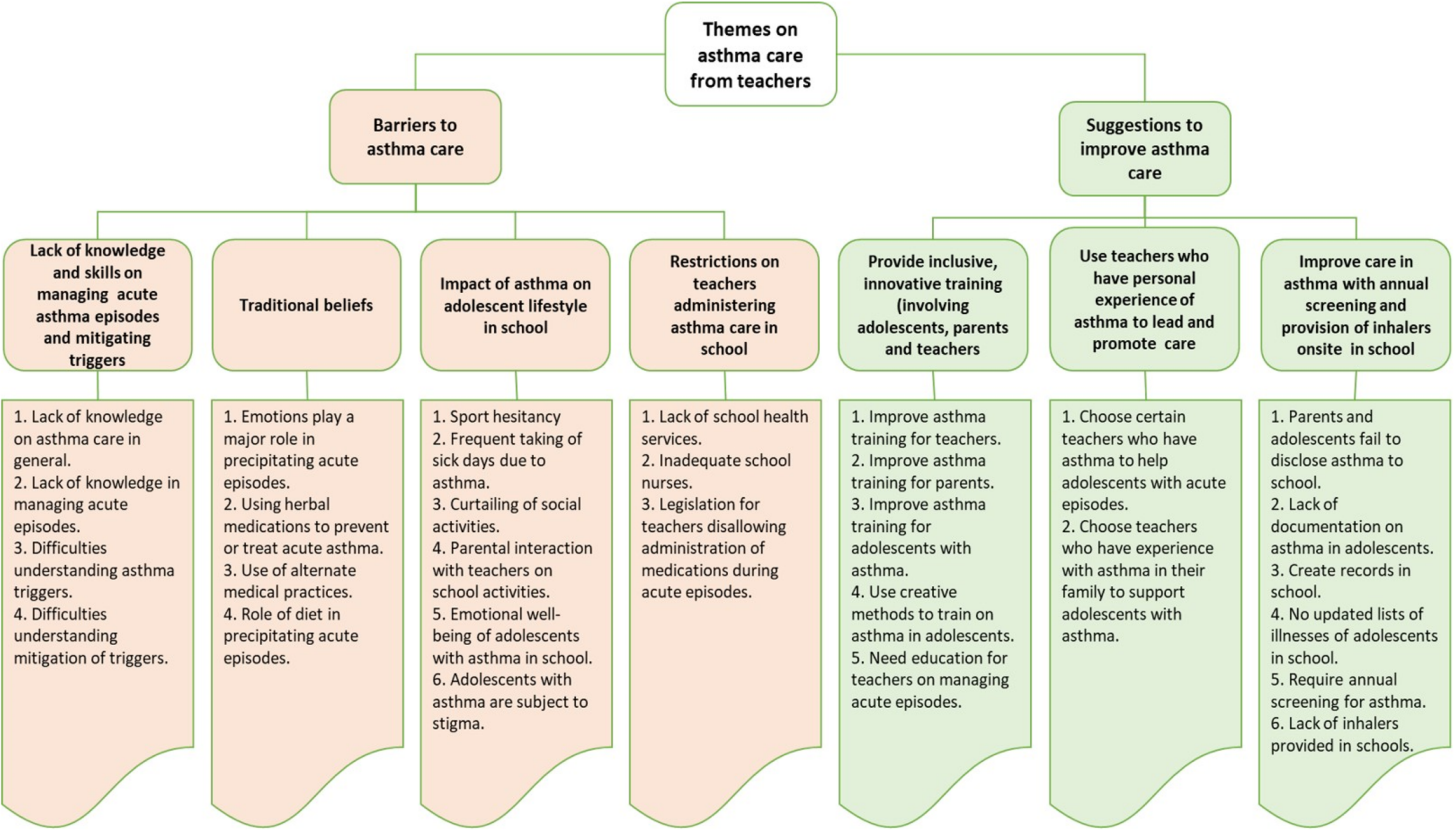
Coding tree and relationships of the various themes identified. Themes on asthma care dervied from the data includes major themes barriers to asthma care and suggestions to improve asthma care with associated sub-themes and their respective minor themes indicated in each panel.

Braun and Clarke’s six-step thematic analysis was then used as the analytic framework to identify the patterns and themes within the coded data^20^. Themes were initially identified based on those commonly occurring codes identified in the transcripts in all countries. The minor themes were highlighted and grouped into sub-themes based on commonly occurring patterns and relationships noted between the identified codes. The various sub-themes were then grouped into the major themes. The coding and theme identification were iterative and involved all members, and consensus was achieved after multiple iterations (Fig. 1 includes the coding tree used to identify the themes (minor themes, sub-themes, and major themes) after analysis. Quotes derived from the data were chosen to illustrate these themes and were specifically selected if they resonated across all countries.

### Patient and public involvement

The FGDs were part of the wider ACACIA study and its patient and public involvement^17^. The FGDs set up, especially for teachers, was developed at each site with the local school staff to provide a friendly and safe experience in a familiar environment. Before other sites, we piloted the questions used in the FGDs in Nigeria to guide the study’s design. Adolescents and teachers were involved at several stages of the ACACIA study but not directly in the recruitment and conduct of the FGDs. A summary of all ACACIA study results will be disseminated to the participating schools, which will communicate it to teachers, adolescents, and parents.

### Reporting summary

Further information on research design is available in the Nature Research Reporting Summary linked to this article.

## Ethics

Ethical approval was granted at all the study sites: Ghana, CHRPE/AP/074/19 and CHRPE/AP/071/20; Malawi, COMREC/P.10/18/2494; Nigeria, LREC/06/10/1084; Uganda, MHREC/1514, and UNCST/SS4940; Zimbabwe, MRCZ/A/2415; South Africa, BREC/BF002/19. Participants provided written informed consent in accordance with both country/local regulations and United Kingdom (UK) ethics regulations. Trial registration number: 269211. Data were de-identified and stored on a password-protected computer with access only to researchers.

## Results

### Study population

The data were transcribed and analysed from 20 FGDs across six countries. A total of 153 teachers were included as study participants, averaging between seven and eight participants per FGD (Table 2). Of these teacher participants (*n* = 153), 66, 7% (*n* = 102) self-identified as female, 55.9% (*n* = 84) knew someone with asthma, 11.1% (*n* = 17) suffered themselves from asthma, and 29.4% (*n* = 45) indicated that they had had formal training in asthma.

**Table 2:** Numbers and distribution of participants in the FGDs across countries.

Country (code)	Number of FGDs conducted	Total number of participants	Percentage of total participants
Ghana (GH)	4	33	21.6
Malawi (MW)	3	10	6.5
Nigeria(NG)	2	24	15.7
South Africa (ZA)	7	57	37.2
Uganda (UG)	2	15	9.8
Zimbabwe(ZA)	2	14	9.2
Totals	20	153	100

FGD designated by country code and number of FGD in that country.

### Identified themes

After coding the data, multiple themes were identified that related to teacher perspectives on asthma care in school. Those themes were found to be common across all countries and were then selected and categorised as minor -themes. Investigators then clustered these 30 minor themes into seven sub-themes that reflected aspects of asthma care within schools. Minor themes had to resonate around a common aspect of care that affected teachers’ perspectives on managing adolescents with asthma in the school for these to be aggregated into a sub-theme. This iterative process focused on perspectives considered most influential on asthma care in all countries. These sub-themes were only finalised when all investigators from all countries had reached a consensus. Investigators’ perspectives then focused these sub-themes into major themes based on the overall aim of the study to determine factors either enabling better care or creating barriers to asthma care within schools. The two major themes were categorised as (1) barriers to caring for adolescents with asthma in school and (2) suggestions to improve the care of adolescents with asthma in school. Figure 1 illustrates the inter-relationship of the minor themes, sub-themes and major themes identified.

Within the first major theme, multiple sub-themes were identified: lack of knowledge and skills on mitigating asthma triggers and managing an acute asthma episode; traditional beliefs – this was defined as commonly held understandings by teachers on causes and management of asthma that are different than those understandings generally accepted within the allopathic understanding of asthma; Impact of asthma on adolescents (sick days, sport hesitancy, stigma from other adolescents, parental denial) and restrictions on teachers administering asthma care in school. In the second theme, the following subthemes were identified: providing inclusive asthma training (involving adolescents, caregivers, and teachers); appointing teachers who have personal experience with asthma to lead and promote care for adolescents with asthma in schools, and improving care for adolescents with asthma with annual asthma screening and provision of inhalers onsite in school.

### Theme 1: Barriers to caring for adolescents with asthma

Lack of knowledge, skills and support in mitigating asthma triggers and managing acute asthma episodes was a universal finding in all FGDs. Teachers identified deficiencies in their knowledge and skills regarding asthma. This lack of knowledge was primarily attributed to a ‘*lack of formal training’* on asthma care in schools and created a general ‘*need* (for) *more information at school level, especially in relation to the technique issues.’* (ZA, FGD 1).

Teachers were particular about which aspects of knowledge and skills in asthma they perceived themselves, the adolescents and their families to have deficiencies in. The deficiencies were related to an inability to adequately care for adolescents during acute asthma episodes and to mitigate against asthma triggers, (Table 3, quotes 1 and 2). This lack of adequate knowledge influenced teachers’ experiences when managing adolescents with acute asthma attacks. Teachers experienced these episodes as *‘daunting’* and *‘scary’*. Furthermore, teachers observed that adolescents were unaware of their condition as asthma or of the severity. There was also a lack of adequate understanding of the role of triggers (exercise, sporting activities, smoke, and dust) and how avoidance of these should be navigated within the school environment.

**Table 3:** Theme 1 Barriers to caring for adolescents with asthma in school.

Sub- theme	Relevant quote
Lack of knowledge and skills on mitigating asthma triggers and managing an acute asthma episode	**Quote 1,** **Mitigating triggers***: “If we have knowledge on asthma and if we know learners with asthma, we can also educate the learners on how to dress for the environment and even the type of ventilation needed, so they will not have a crisis. So, all this boils down to us having education or training…”* (GH, FGD 1) **Quote 2,** **Managing an acute attack***: “It’s best to make sure that we are dealing with a correct problem first. Make sure that it’s an asthmatic attack. You may think it’s an asthmatic attack and when it’s something else. So, I think it’s best to ask whether the child has had the attack before then from there, how is it handled? Maybe the child knows. Maybe the child has got an inhaler ….”* (ZW, FGD 1)
Traditional beliefs on asthma	**Quote 3,** **Religious**: “*Asthma is a contagious disease. It is a demonic condition.”* (GH, FGD 2) **Quote 4,** **Use of Herbs:** “*If there is an herbalist nearby. That person can help us to supress the condition and the person can get better. So, some of these things we should not look down or shun them. Help is help, and we will be needing help*.”(ZW, FGD 1) **Quote 5,** **The role of emotions in an acute episode:** *“When you ‘bark’ at them, they can tend to collapse, whether it is real or pretending, but they use that … This affects their performance in all matters, be it academic, and be it social, they want to have a special consideration in everything that is done.”*(UG, FGD 2) **Quote 6**, “*When the child is growing up and keeps having asthma frequently, his confidence starts to go away… To the extent that even when it’s time to write exams, he gets into the exam room and starts getting sick. When the exam is over, he would be okay.”*(MW, FGD 1)
Impact of asthma on adolescents (sick days, sport hesitancy, stigma from other adolescents, parental denial)	**Quote 7, Sick day**, “*For instance, a child in my school (an orphan who lives with his grandmother) whenever he has attack, he doesn’t come to school….”* (NG,FGD 2) **Quote 8, Sport hesitancy**. “*Students in primary school are energetic and enjoy running around the place and playing. So now imagine a student with asthma, they would run a bit and the next thing an asthma attack strikes. That means the learners cannot engage in activities being done by his/her age mates.”*. (ZW, FGD 2) **Quote 9, Stigma:** “*Some are shy and do not want to tell others, but some who know quite well that they have asthma, they like to hide it….As students* (adolescents without asthma) *most often stigmatised such asthmatic patients. For instance, one of the students may tell others not to sit behind or near one who has asthma in the classroom.”* (NG, FGD 1)
Restrictions on administering asthma care in school	**Quote 10, Lack of school health services**: *“school nurses who have ‘stopped completely and do nothing now’ or just ‘give the vaccine and … are gone and … are not seen.”*(ZA, FGD 4) **Quote 11, Restriction on teachers providing medical care:** “*Government policy is, that we dare not buy or give a child any medication. Lagos State policy says if any child falls sick in the school you ought to take the child to the nearest health centre.”* (NG, FGD 2)

The most common traditional beliefs held were related to the dominant role emotions play in triggering asthma attacks and the use of alternative treatments for asthma, e.g., traditional herbs, (Table 3, quote 4). There were also various religious beliefs on the causes and potential cures for asthma. Attributing a religious aetiology to asthma was predominant in all FGDs across the countries but noticeably absent from South African FGDs, (Table 3, and quote 3). Within this sub-theme, teachers identified emotions as a major trigger for acute asthma episodes in schools. However, they also believed that some reported asthma episodes among adolescents were indeed more emotional outbursts than true asthma symptoms, (Table 3, quote 5). Teachers’ reflected on the impact that uncontrolled asthma had on various learning activities. These included taking frequent sick days and sports hesitancy, leading to many adolescents not participating in scheduled school and physical activities, (Table 3, quotes 7 and 8). Sport and physical activity hesitancy were noted with ‘*some of those parents who do not allow the kids to even participate in sport’*, and parents are seen to ‘*molly-coddle’* or be ‘*overprotective*’. The impact of parental anxiety leads to adolescents ‘*who shy away from participating because they believe that’s what they feel*’. (ZA, FGD 4). Stigma associated with having an asthma diagnosis among adolescents was included in this sub-theme. Having asthma was stigmatising among adolescents with asthma and was associated with denial and failure to disclose the diagnosis to teachers, thereby delaying or preventing proper management, (Table 3, quote 9). The aspects noted in this sub-theme that impacted asthma care, included the limited scope of school health services in most African countries and the prohibition of teachers from administering medicines without parental consent. Regarding school health services*,* teachers noted that school nurses mostly focused on immunisation programmes and had little to do with health screening or first-line management of medical problems, (Table 3, quote 10). Secondly, teachers were not empowered by any legislation to administer medicines without parental consent. These restrictions make many teachers feel less comfortable giving adolescents’ inhalers when needed, (Table 3, quote 11).

### Theme 2: Suggestions to improve the care of adolescents

Teachers suggested using all-inclusive training to improve asthma knowledge in parents, teachers and adolescents, (Table 4, quote 12). A strong willingness and openness to training in asthma were seen where teachers, parents and adolescents could create *‘an environment that is positive for the child to actually feel, if they have asthma, but they can still do exercise, they can still swim, they can still take part in sport because they are comfortable that the people around them are educated enough to know that when they are going to be in danger.’* (ZA, FGD 1). For training and information dissemination to be effective it should be for *‘everyone, for doctors, nurses, parents, learners, so it is not only learners or people that need to be taught about it…’* (ZA FGD 3). Teachers indicated that parents and adolescents must be involved in proposed asthma education for schools, emphasising an inclusive approach in these programmes. Using creative methods such as drama (plays) and music to educate adolescents, specifically on asthma, was seen as important as the training content, particularly to sustain learner interest, (Table 4, quote 13).

**Table 4:** Theme 2 Suggestions by teachers to improve the care of adolescents with asthma in school.

Sub-theme	Relevant quote
Inclusive, innovative asthma training needed (involving adolescents, caregivers, and teachers)	**Quote 12, Inclusive training**: “*I think asthma should be brought on the red list of health challenges of learners, and the approaches to handling issues of that kind be known to every person, that is very important.”* (UG, FGD 2) **Quote 13,** **Innovative training**:“*The students are normally enthusiastic they really want to participate, sometimes it might be a drama, a play let, even from the assembly you know some come with banners, you talk about it on that day, you carry everybody along, the teachers can even organise and bring in parents as well.”* (ZA,FGD 2)
Appoint teachers who have personal experience with asthma to lead and promote care for adolescents with asthma in schools	**Quote 14*, “****And if they* (referring to adolescents with asthma) *have a teacher that is experiencing the same thing, they’d understand that my teacher is going through the same thing. If I’m having a problem, I can go to them* (referring to those teachers with asthma)*, I can speak to them*.” (ZA, FGD 6) **Quote 15*, “****It would help if you could maybe get into schools and select a few staff members that you can train and impart knowledge on asthma as well as give them pamphlets that will aid. This will actually come in handy let’s say for example if myself as a teacher I come across such a situation in my class, I am in a position to rush to the trained teacher to ask for help and the student will be assisted faster and appropriately.”* (ZW, FGD 2)
Improving care for adolescents with asthma with annual asthma screening and provision of inhalers onsite in school	**Quote 16, Annual Screening: “**Screening *at the beginning of the year by the Department of Health … as well as once a year and do a screening. Or even like maybe in Grade 1 and then in Grade 4 again, different phases so as time goes you know whether the child has developed it* (referring to asthma) or *not.”* (ZA,FGD 7) **Quote 17, Inhalers in residential schools:** “*I just think that as a school, if we had a facility like in the staff room where we have inhalers … that by the time we are calling the principal or boarding masters, we already have these things. Because these kids will sometimes run out of their medication and won’t say anything*.”(MW, FGD 1)

Teachers expressed a view that their colleagues with personal experiences with asthma (either being asthma sufferers themselves or caring for close members of their family with asthma) had a better understanding of how to relate to adolescents with asthma than teachers with no such experience, (Table 4, quotes 14 and 15). Teachers’ across most countries expressed concern that annual screening for asthma and other chronic diseases was not uniformly performed across all schools. They supported the interventions of annual health screening for common conditions, including asthma, which should include disclosing medical conditions by all adolescents. This would allow teachers to be aware of potential challenges with the adolescents during the year, (Table 4, quote 16). Teachers also indicated that providing metered dose inhalers in all schools, especially in residential (boarding) schools, would assist adolescents having acute asthma episodes, (Table 4, quote 17).

## Discussion

Our study has identified teachers’ perspectives on barriers to caring for adolescents living with asthma and their suggestions for modifying specific practices within schools to improve asthma care across six sub-Saharan African countries. These barriers and suggestions on asthma care were common to all countries, with little variation between them.

The perceived deficit in asthma knowledge expressed by teachers across our study has been frequently documented across urban, rural and low-resourced schools worldwide^21–23^. This lack of knowledge has been postulated to contribute to poor self-confidence and self-efficacy among teachers, especially when managing adolescents with acute asthma episodes^13,14^. The overall goal in asthma care is the provision of a ‘seamless blanket of care’ across home, school and health facilities, but that is compromised when teachers lack basic skills and knowledge for caring for adolescents with asthma^9,11,24^. This study identified that for effective improvement in asthma knowledge and care, all stakeholders (adolescents, parents and teachers) require exposure to training programmes. This need for all-inclusive training seems highly recommended for the sSA context, where parents often lack adequate knowledge and resources to manage asthmatic adolescents outside the school environment. In facilities with a limited healthcare workforce and multiple priorities, asthma training devolved to the school environment may offer a solution to improving asthma knowledge. This finding may also explain studies that have documented that while isolated asthma training for teachers in schools improves teacher knowledge, the overall impact on asthma care following this training remains guarded^15,16^. Thus, an inclusive approach should be prioritised as a model for comprehensive asthma education in schools. An all-inclusive asthma training approach may remedy low levels of ‘health literacy’ (‘defined as an “individual’s capacity to access, understand, communicate, utilise, and make decisions based on health information”) that exacerbate anxiety felt by teachers^22^. The emphasis on developing ‘health literacy’ among adolescents, their caregivers and teachers can potentially improve overall asthma control of children and adolescents^25,26^.

A tailored approach includes focusing on the practical aspects of using metered-dose inhalers during an acute episode and mitigating asthma triggers in the school environment. Similar findings have been reflected in studies that show knowledge deficiencies among teachers relate specifically to the technical aspects and fear of overdosing when using inhalers^23,24^.

Teachers further suggested alternative methods compared to traditional lecture formats would be more successful in sustaining interest in asthma education. Creative modalities, like drama and music involving adolescents and their parents, have the potential to address misconceptions leading to frequent school absences, restrictions on sporting activities and the stigma related to asthma^21^.

The call for selecting teachers with personal experiences in dealing with asthma as preferred ‘asthma champions’ has been documented globally and is a potential strategy to improve asthma outcomes^15,27,28^. The creation of ‘asthma champions’ amongst certain school teachers can help bridge the critical gap in advocacy. ‘Asthma champions’ improve self-confidence and self-efficacy among teachers and promote communication among healthcare providers, parents, teachers and community support groups^27^. This suggestion in our study addresses the lack of nurses in African schools. It strengthens the role of teachers within communities where health education and advocacy are often lacking for non-communicable diseases. Another advantage of teachers being ‘asthma champions’ is their potential role in dissipating certain beliefs that hinder acute and preventative asthma care within low-resource contexts^29^.

School health systems should make provisions for screening and documenting adolescents with asthma and clarify the roles of teachers in acute care. Teachers in this study reiterated formalising annual health screening with record keeping to identify adolescents with asthma or asthma symptoms. Information gathered could assist with potential acute asthma episodes and the possible need for trigger avoidance measures amongst adolescents. Record keeping with action plans in school healthcare systems has been shown to improve staff preparedness and decrease anxiety^16^.

Multiple concerns were raised regarding the lack of clarity with legislation restricting teachers from administering medication to adolescents without parental approval. In the context of inadequate provision of on-site school nurses noted in many LMICs, the calls for clear policy guidelines on the role of teachers in administering asthma treatment in acute episodes are crucial^30^.

This study’s strength is drawing on multiple African countries to comprehensively assess teachers’ perspectives in these LMICs. Most studies in Africa have been single-centre or in one country, limiting the inference to the diversity of countries across the continent. This study has some limitations as it was mainly focused on urban-based schools with no rural representation. We did not conduct member checks. We used an inductive approach with constant data comparisons and discussions to inform our understanding of the views expressed in the FGDs. We were aware of the risk of researcher bias in qualitative research and actively discussed reflexivity in the regular multidisciplinary team discussions, which aided analysis and informed interpretation.

With increasing asthma rates across sSA, a more significant role for teachers is being identified in managing asthma in adolescents. Barriers include a perceived lack of skills and knowledge and traditional beliefs about asthma. Asthma education is suggested for adolescents, their caregivers and teachers who use innovative methods focused on improving asthma knowledge and dispelling myths. Additionally, annual health screening and using teachers as ‘asthma champions’ who advocate for adolescents with asthma may assist in managing this common non-communicable condition.

### Supplementary information

Supplementary Information File

Reporting Summary

## Data Availability

Anonymised data is available from the corresponding author upon request, and there are no restrictions to data usage.
