# Supplementary material for: A cross-country qualitative analysis of teachers’ perceptions of asthma care in sub-Saharan Africa
Source: NPJ Prim Care Respir Med. 2023 Sep 23;33:31. doi: 10.1038/s41533-023-00354-7 (PMC10517916; doi:10.1038/s41533-023-00354-7)
Supplement: Supplementary file 1 — Supplementary Information File [file 41533_2023_354_MOESM1_ESM.pdf]

(Supplementary Information file). Final codebook: Teachers' perceptions of asthma care in Sub-Saharan Africa: A cross-country qualitative analysis

**Consensus code book developed to evaluate each transcript**		
**Code**	**Definition**	**Purpose/meaning of code**
1. knowledge of asthma	Participant defines asthma/ describe asthma symptoms	Indicates participant's knowledge of what asthma is
2. Beliefs	Teachers own beliefs on what asthma is	States teacher's personal belief on asthma this is not in asthma management guidelines
3. Experience of asthma in school	Participant recalls or describes an experience they have had when an adolescent had an asthma attack in their class	Try to find out their response to an emergency, what measures are in place to help a learner with asthma
4. Experience of asthma outside of school	Participant recalls or describes their experience with an asthmatic person	if they know anyone with asthma., presentation, what was done, Also try and find out their perceptions of people with asthma

(Supplementary Information file). Final codebook: Teachers' perceptions of asthma care in Sub-Saharan Africa: A cross-country qualitative analysis

5. Care for asthmatic adolescent	Participant describes what they would need in order to attend or help an asthmatic in class	Try to find out what access or information they need to better take care of an adolescent with asthma
6. Effects of asthma on schooling	Participant describes from their experience how asthma has affected the learning process of the student	Try to find out how asthma has affect the learning in terms of their education , absenteeism, participation in social activities – sports, behaviour and relationship with other students
7. Enablers to asthma care in school	Participant suggest how asthmatic learners lives can be made better inside and outside of school	Try to find out what can be done by school, parents, and teachers, healthcare to improve the life of an asthmatic adolescent. What kind of tools or information needs to be available
