# Supplementary material for: A cross-country qualitative analysis of teachers’ perceptions of asthma care in sub-Saharan Africa
Source: NPJ Prim Care Respir Med. 2023 Sep 23;33:31. doi: 10.1038/s41533-023-00354-7 (PMC10517916; doi:10.1038/s41533-023-00354-7)
Supplement: Supplementary file 2 — Reporting Summary [file 41533_2023_354_MOESM2_ESM.pdf]

nature portfolio

Corresponding author(s): Dr Kimesh Loganathan Naidoo

Last updated by author(s): Sep 7, 2023

Reporting Summary

Nature Portfolio wishes to improve the reproducibility of the work that we publish. This form provides structure for consistency and transparency in reporting. For further information on Nature Portfolio policies, see our Editorial Policies and the Editorial Policy Checklist.

Statistics

For all statistical analyses, confirm that the following items are present in the figure legend, table legend, main text, or Methods section.

n/a	Confirmed	
☒	☐	The exact sample size (*n*) for each experimental group/condition, given as a discrete number and unit of measurement
☒	☐	A statement on whether measurements were taken from distinct samples or whether the same sample was measured repeatedly
☒	☐	The statistical test(s) used AND whether they are one- or two-sided<br>*Only common tests should be described solely by name; describe more complex techniques in the Methods section.*
☒	☐	A description of all covariates tested
☒	☐	A description of any assumptions or corrections, such as tests of normality and adjustment for multiple comparisons
☒	☐	A full description of the statistical parameters including central tendency (e.g. means) or other basic estimates (e.g. regression coefficient) AND variation (e.g. standard deviation) or associated estimates of uncertainty (e.g. confidence intervals)
☒	☐	For null hypothesis testing, the test statistic (e.g. *F*, *t*, *r*) with confidence intervals, effect sizes, degrees of freedom and *P* value noted<br>*Give P values as exact values whenever suitable.*
☒	☐	For Bayesian analysis, information on the choice of priors and Markov chain Monte Carlo settings
☒	☐	For hierarchical and complex designs, identification of the appropriate level for tests and full reporting of outcomes
☒	☐	Estimates of effect sizes (e.g. Cohen's *d*, Pearson's *r*), indicating how they were calculated

Our web collection on statistics for biologists contains articles on many of the points above.

Software and code

Policy information about availability of computer code

Data collection	No software was used
Data analysis	NVivo 1.6 software program

For manuscripts utilizing custom algorithms or software that are central to the research but not yet described in published literature, software must be made available to editors and reviewers. We strongly encourage code deposition in a community repository (e.g. GitHub). See the Nature Portfolio guidelines for submitting code & software for further information.

Data

Policy information about availability of data

All manuscripts must include a data availability statement. This statement should provide the following information, where applicable:

- Accession codes, unique identifiers, or web links for publicly available datasets
- A description of any restrictions on data availability
- For clinical datasets or third party data, please ensure that the statement adheres to our policy

Anonymised data is available from the corresponding author upon reasonable request

Research involving human participants, their data, or biological material

Policy information about studies with human participants or human data. See also policy information about sex, gender (identity/presentation), and sexual orientation and race, ethnicity and racism.

Reporting on sex and gender	Of these teacher participants (n=120) 67.5% self identified as female, 65% knew someone with asthma, 13.3% suffered themselves with asthma and only 34.2% indicated that they had any formal training with asthma.
Reporting on race, ethnicity, or other socially relevant groupings	No participant data on race or ethnicity was collected. They study was geographically situated across the continent of Africa.
Population characteristics	This included teachers at the three schools selected in each city involved in the ACACIA project. The teachers selected to participate in this study were drawn from those schools where both adolescents with a doctor's diagnosis of asthma and symptoms of asthma but without a diagnosis were identified. A convenience sample of teachers who consented to be included in the study were the study participants.
Recruitment	The teachers selected to participate in this study were drawn from those schools where both adolescents with a doctor's diagnosis of asthma and symptoms of asthma but without a diagnosis were identified. A convenience sample of teachers who consented to be included in the study were the study participants.
Ethics oversight	Ethical approval was granted at all the study sites: Ghana, [name deleted to maintain the integrity of the review process] and [name deleted to maintain the integrity of the review process]; Malawi, [name deleted to maintain the integrity of the review process]; Nigeria, [name deleted to maintain the integrity of the review process]; Uganda, [name deleted to maintain the integrity of the review process], and [name deleted to maintain the integrity of the review process]; Zimbabwe, [name deleted to maintain the integrity of the review process]; South Africa, [name deleted to maintain the integrity of the review process]. Participants provided written informed consent in accordance with both country/local regulations and United Kingdom (UK) ethics regulations. Trial registration number (name deleted to maintain the integrity of the review process). Data were de-identified and stored on a password-protected computer with access only to researchers.

Note that full information on the approval of the study protocol must also be provided in the manuscript.

Field-specific reporting

Please select the one below that is the best fit for your research. If you are not sure, read the appropriate sections before making your selection.

☐ Life sciences ☒ Behavioural & social sciences ☐ Ecological, evolutionary & environmental sciences

For a reference copy of the document with all sections, see nature.com/documents/nr-reporting-summary-flat.pdf

Behavioural & social sciences study design

All studies must disclose on these points even when the disclosure is negative.

Study description	Cross-sectional qualitative
Research sample	School teachers: This included teachers at the three schools selected in each city involved in the ACACIA project. The teachers selected to participate in this study were drawn from those schools where both adolescents with a doctor's diagnosis of asthma and symptoms of asthma but without a diagnosis were identified. A convenience sample of teachers who consented to be included in the study were the study participants.
Sampling strategy	A convenience sample of teachers who consented to be included in the study were the study participants
Data collection	Focus groups were conducted face-to-face, with adherence to in-country-specific COVID-19 regulations. All FGDs were moderated by at least two facilitators, one of whom was a study investigator at each site. FGDs took place in English. Focus groups were audio-recorded and transcribed verbatim. Discussions lasted between 40-60 minutes and followed a pre-defined question guide specific to each focus group category (Table 1). The ACACIA core study team developed the discussion guide after multiple iterations. The underlying framework that informed the development of the discussion guide by this team was a literature review on barriers to asthma care amongst children with asthma worldwide 17.
Timing	between 01 November 2020 and 30 June 2021
Data exclusions	No data was excluded
Non-participation	No participants were excluded
Randomization	participants were not randomised into groups

Reporting for specific materials, systems and methods

We require information from authors about some types of materials, experimental systems and methods used in many studies. Here, indicate whether each material, system or method listed is relevant to your study. If you are not sure if a list item applies to your research, read the appropriate section before selecting a response.

Materials & experimental systems

n/a	Involved in the study
☒	☐ Antibodies
☒	☐ Eukaryotic cell lines
☒	☐ Palaeontology and archaeology
☒	☐ Animals and other organisms
☒	☐ Clinical data
☒	☐ Dual use research of concern
☒	☐ Plants

Methods

n/a	Involved in the study
☒	☐ ChIP-seq
☒	☐ Flow cytometry
☒	☐ MRI-based neuroimaging
